# Contrasting Complement Control, Temporal Adjunct Control and Controlled Verbal Gerund Subjects in ASD: The Role of Contextual Cues in Reference Assignment

**DOI:** 10.3389/fpsyg.2017.00448

**Published:** 2017-03-28

**Authors:** Vikki Janke, Alexandra Perovic

**Affiliations:** ^1^Department of English Language and Linguistics, Rutherford College, School of European Culture and Languages, University of KentCanterbury, UK; ^2^Division of Psychology and Language Sciences, Department of Linguistics, University College LondonLondon, UK

**Keywords:** autism, syntax, pragmatics, control, language development, language impairment

## Abstract

This study examines two complex syntactic dependencies (complement control and sentence-final temporal adjunct control) and one pragmatic dependency (controlled verbal gerund subjects) in children with ASD. Sixteen high-functioning (HFA) children (aged 6–16) with a diagnosis of autism and no language impairment, matched on age, gender and non-verbal MA to one TD control group, and on age, gender and verbal MA to another TD control group, undertook three picture-selection tasks. Task 1 measured their base-line interpretations of the empty categories (*ec*). Task 2 preceded these sentence sets with a weakly established topic cueing an alternative referent and Task 3 with a strongly established topic cueing an alternative referent. In complement control (Ron persuaded Hermione *ec* to kick the ball) and sentence-final temporal adjunct control (Harry tapped Luna while *ec* feeding the owl), the reference of the *ec* is argued to be related obligatorily to the object and subject respectively. In controlled verbal-gerund subjects (VGS) (*ec* Rowing the boat clumsily made Luna seasick), the *ec*'s reference is resolved pragmatically. Referent choices across the three tasks were compared. TD children chose the object uniformly in complement control across all tasks but in adjunct control, preferences shifted toward the object in Task 3. In controlled VGSs, they exhibited a strong preference for an internal-referent interpretation in Task 1, which shifted in the direction of the cues in Tasks 2 and 3. HFA children gave a mixed performance. They patterned with their TD counterparts on complement control and controlled VGSs but performed marginally differently on adjunct control: no TD groups were influenced by the weakly established topic in Task 2 but all groups were influenced by the strongly established topic in Task 3. HFA children were less influenced than the TD children, resulting in their making fewer object choices overall but revealing parallel patterns of performance. In this first study of three sub-types of control in ASD, we demonstrate that HFA children consult the same pragmatic cues to the same degree as TD children, in spite of the diverse pragmatic deficits reported for this population.

## Introduction

If a lay person is asked to consider which aspect of communication causes most difficulty to individuals with autism spectrum disorder (ASD), first and foremost, their thoughts will go to that aspect of language that uses context and real world knowledge to establish intended meanings, known in the linguistics field as pragmatics. Indeed, when summarizing the principal language problem in ASD, current textbooks continue to describe pragmatics as being the most pervasive, whilst slowly recognizing that varying syntactic deficits occur in this heterogeneous population, too (see Cummings, 2016). The term pragmatics, however, is used to cover an enormous range of skills, including the ability to understand non-literal meanings, such as those used in metaphor, irony and humor (see Ozonoff and Miller, 1996; Dennis et al., 2001; MacKay and Shaw, 2004; Martin and McDonald, 2004; Norbury, 2005; Rundblad and Annaz, 2010), the socially-based ability to listen and respond appropriately in conversational exchanges (see Tager-Flusberg and Anderson, 1991; Boucher, 2009) but also knowledge of how to make use of contextual information when encountering sentences that have more than one interpretation. This can include resolution of a structural ambiguity, such as in (1), where depending on the attachment site of the adjunct, either argument could be understood as being in possession of the stick. Alternatively, the choice might stem from a referential under-specification, as in (2), where the agent of the infinitival verb in the bracketed clause could be equated with the sentential argument (i.e., *Luna*), or someone else entirely, even if in the absence of further context, we are first drawn toward the so-called “sentence-internal referent” interpretation.

(1) The angry man chased the boy with a big stick.
(2) [Rowing the boat clumsily] made Luna seasick.

On the basis of these few examples, we can see that a very broad range of skills are covered by this umbrella term. Often, a distinction is drawn between complex pragmatic tasks that require a person to go beyond the literal meaning, such as in irony, and those that only require one to reach a literal meaning that is contextually determined, as seen in reference assignment. The former is sometimes referred to as secondary pragmatics and the latter as primary (see Recanati, 2004). It is on the latter type that this study focuses, together with syntactic competence. We compare the degree to which typically developing (TD) children and children diagnosed as HFA consult contextual cues when interpreting sentences that contain an underspecified term, whose reference depends upon another, fully specified term. This fully specified term may be in the same sentence, in which case it is a linguistic antecedent, but it may also occur outside of the sentence, in which case it is a discourse antecedent. Our aim is to establish if attendance to contextual cues differs in these populations when they engage in the task of reference assignment.

The sentences we focus on are called control constructions, which include a range of sentences whose interpretations are regulated syntactically or pragmatically. Prototypical examples of two sub-types of syntactically regulated control can be seen in (3) and (4), where in both cases the interpretation of the understood agent (represented as *ec* for empty category) is restricted to a unique interpretation (see Williams, 1980; Landau, 2013). In (3), which is an example of complement control (CC), the agent of the verb in the complement clause must be the matrix object (i.e., *Hermione*), whereas in (4), which is an example of sentence-final temporal adjunct control (AC), the agent of verb in the adjunct clause is interpreted as the matrix subject (i.e., *Harry*) by most people.

(3) Ron persuaded Hermione [*ec* to kick the ball].
(4) Harry tapped Luna [while *ec* feeding the owl].

The syntactic nature of the relation between the antecedent (i.e., the element which controls the *ec*'s interpretation) and the *ec* in complement control becomes clear if we illustrate the sentential restrictions on the *ec*'s interpretation. Example (5) shows that the antecedent must come from within the sentence, that it must be local, and that it needs to be higher in the structure than the *ec* (see Williams, 1980; Manzini, 1983; Hornstein, 2001). In (5a), for example, only *Hermione* can be interpreted as the agent of *kick*. The indices also show that a sentence-external referent is not permitted and that the subject, though sentence-internal, cannot control the *ec*, it not being the most local contender^1^. (5b) demonstrates the structural superiority requirement, where only *Hermione's cousin* (and not *Hermione*) can be the *ec*'s antecedent, since only the whole possessive DP c-commands into the infinitival clause.

(5) a. Ron_i_ persuaded Hermione_*j*_ [*ec*_i/^*^j/^*^k_ to kick the ball].
     b. Ron persuaded Hermione's cousin_i_ [*ec*_i_ to kick the ball].

The *ec* in sentence-final temporal adjunct control has long been reported as similarly restricted in terms of the syntactic antecedent it can take. It does not permit external referents, as shown in (6a), and its antecedent must also c-command it, as illustrated in (6b). (6a) also suggests that an object-oriented reading of the *ec* is barred. The adjunct, not having been selected by the matrix verb, is free to attach high, where only the subject c-commands it (see Landau, 2013). This high attachment also makes the subject the most local.

(6) a. Harry_1_ tapped Luna_2_ [while [*ec*_1/^*^2/^*^3_ reading the book]].
     b. Harry's cousin_1_ tapped Luna [while [*ec*_1_reading the book]].

In juxtaposition to these syntactically regulated examples of control are pragmatically regulated ones, which admit variable reference. In the controlled verbal-gerund subject (VGS) below, which we met first in (2) above, the agent of the verb could be the sentential argument or someone else, although in the absence of context, the sentential argument is the preferred choice of most child and adult speakers (see Janke, 2016; Janke and Bailey, 2017 respectively). The fact that this example also permits an external-referent reading demonstrates the absence of the syntactic restrictions we saw for complement and sentence-final temporal adjunct control above.

(7) [*ec*_?_ Rowing the boat clumsily] made Luna seasick.

Typically developing children start producing complement control sentences quite early, namely from 3 years, but comprehension studies have shown that for a short while after this, their interpretation of the *ec* is not fixed (Eisenberg and Cairns, 1994). From about 5–6 years, however, the majority of children restrict their interpretations in complement control to the object, even in the presence of pragmatic leads that cue subject interpretations (Lust, 1987; Cohen Sherman and Lust, 1993; Janke, 2016). This is in contrast to their interpretations of overt pronouns, for example, for which they do consult leads when determining who the pronouns refer to (see Cohen Sherman and Lust, 1993). Sentence-final temporal adjunct control occurs in production later than complement control (see Broihier and Wexler, 1995) and for a few years, some children accept subject, object and external-referent interpretations of them (see Lust et al., 1986; McDaniel et al., 1990/1991; Goodluck and Behne, 1992). However, by the age of about seven, non-subject interpretations are very rare in the absence of pragmatic leads (see Hsu et al., 1989). More recently, Janke (2016) demonstrated that children aged between 6;9 and 11;3 do in fact permit object interpretations when that object is cued by a strongly established topic. The same result was found in a comparable study on 70 adults (Janke and Bailey, 2017). They did not, however, accept external-referent interpretations under the same amount of discourse pressure. This is important as it demonstrates that the fragility of this particular sub-type of adjunct control is restricted to sentence-internal arguments and so not to be confused with a pragmatically regulated control relation, such as controlled VGSs. These constructions have been studied less, but those that exist report mixed results. Adler (2006) and Goodluck (1987), using a truth-value judgment task and an act-out task respectively, found a preference for the sentence-external referent in children under six. In contrast, Janke (2016), which used a picture-selection task, reported that children from six onwards demonstrated a strong preference for sentence-internal referent interpretations, a preference which could be altered when the critical sentences were cued with pragmatic leads. There is a variability, however, in children's and adults' referent choices in these constructions, which is expected in a pragmatically regulated relation.

An interesting question regarding these three sub-types of control and (language in) ASD is whether or not children with ASD would converge on the same referential choices that typical populations do, or whether idiosyncrasies in the cognitive profile of individuals with ASD could influence their interpretations of linguistic constructions for which both syntactic and pragmatic proficiency is required. One type of executive function skill, namely that of cognitive flexibility, has been argued to be linked to obsessive and repetitive behaviors in ASD (e.g., South et al., 2007), and possible pragmatic deficits (Kissine, 2012). Deficiencies in cognitive flexibility, or the “ability to shift to different thoughts or actions depending on situational demands” (Geurts et al., 2009, p. 74), could certainly result in different patterns of interpretation of pragmatically controlled control constructions for children with ASD compared to TD controls, though these may not be relevant to interpretation of syntactically regulated constructions, such as complement control and sentence-final temporal adjunct control.

Complement control and temporal adjunct control are syntactically regulated relations that involve a CP-layer (their infinitival clauses are CPs, see Chierchia, 1984) and so are examples of complex syntax. There are mixed results in the literature as to whether children with ASD are fully proficient at this level of grammar. In the first two studies on this construction in autism, Janke and Perovic (2015) and Janke and Perovic (2016) showed that regular complement control caused no interpretative difficulties in two different populations of high-functioning children with autism (HFA). However, other examples of complex syntax may be compromised in this sub-group. Perovic et al. (2007), for example, reported on a group of HFA children having difficulty with raising constructions (i.e., Homer seems to Bart *t* to be wearing a hat), which are traditionally analyzed as instances of A-movement, where the argument is interpreted in a different (argument) position from which it originated. Constructions involving other types of movement (i.e., A-bar movement, where the argument moves to a non-argument position), such as relative clauses, have also been found to cause difficulty in some populations with ASD (Riches et al., 2010; Durrleman et al., 2016). It seems then, that syntactic relations that involve displacement can be compromised in some HFA populations, whereas those that do not are spared (see Janke and Perovic, 2015). Perovic et al. (2013a,b), for example, reported that reflexive binding caused no problems to HFA children classified as ALN (autism with normal language). Reflexive binding is a relation that does not incorporate movement and shares many other syntactic properties with obligatory control (see Manzini, 1983; Koster, 1987; Janke, 2008).

Unlike complement control, the interpretation of controlled VGSs depends heavily on the context. The examples below demonstrate this point. In (8a), as we saw above, there is a strong inclination to interpret the sentence-internal referent as the *ec*'s antecedent. However, this is not fixed, as evidenced by the manner in which our interpretations can change in (8b and c). (8b) provides a “weakly established topic,” in that the introductory sentence promises to make *Ron* the topic of the forthcoming sentence (see Janke and Bailey, 2017). The *ec* in the following sentence is a discourse-anaphoric element so it can take its reference from this weakly established topic. The example in (8c) demonstrates a stronger cue, utilizing a “strongly established topic.” In this example, the first sentence is about *Ron*, thereby making this DP the sentence topic, and the person *Ron* refers to is elaborated on and continues as the topic of discourse in the following sentence. In TD children and adults, these topics are very persuasive. The weakly established topic switches the majority of participants' referent choices toward it and the strongly established topic does so nearly uniformly (Janke, 2016).

(8) a. *ec* Rowing the boat clumsily made Luna seasick.
     b. Let me tell you something about Ron. *ec* Rowing the boat clumsily made Luna seasick.
     c. Ron is taking a trip onto Hogwarts lake. Ron takes hold of the wood oars. *ec* Rowing the boat clumsily made Luna seasick.

Janke and Perovic (2016) tested a group of HFA children on controlled VGSs and found that the children showed a similar level of susceptibility to the pragmatic leads as their control children. This is in contrast to the widely established view that all pragmatics in ASD is deficient: their results suggest that pragmatic skills relevant to the selective and appropriate use of context to decide who is being spoken about in undetermined circumstances are functioning well in this population. Importantly, both the TD and HFA children ignored topics in sentences preceding complement control, as in (9a–c), and so chose the object uniformly.

(9) a. Harry told Luna to pop the balloon.
     b. Let me tell you something about Harry. Harry told Luna to pop the balloon.
     c. Harry is performing a new trick. Harry takes out a pin. Harry told Luna to pop the balloon.

This stable pattern is expected because as the product of a syntactically regulated relation, the *ec* in complement control should resist outside interference, which is exactly what this paradigm revealed. What is not yet known, however, and is a question that the current paper will address, is how HFA children respond to topics that cue the object in temporal adjunct control, as in (10) below.

(10) a. Harry tapped Luna while feeding the owl.
     b. Let me tell you something about Luna. Harry tapped Luna while feeding the owl.
     c. Luna is looking after the birds for the day. Luna takes out the bird seed. Harry tapped Luna while feeding the owl.

This sub-type of control has not been examined in ASD before so by conducting this first analysis on sentence-final temporal adjunct control we can provide an important contribution to the growing portrait of complex syntactic abilities in this population. However, there is another reason for this construction being an interesting topic to examine in children with autism, which relates to work that has revealed a lenience it exhibits in terms of the interpretations its *ec* permits. Recent experimental work on this sub-type of adjunct control has indicated that children's and adults' interpretations of the *ec* are not quite as previously assumed in the literature (see Janke, 2016, for children and Janke and Bailey, 2017, for adults). Using the aforementioned pragmatic lead paradigm, participants were asked to make referent-choice decisions in different sub-types of control which were preceded by no contextual cue, a weak contextual cue or a strong contextual cue. The results revealed temporal adjunct control not to be rigidly subject-oriented. Specifically, although a weak contextual cue toward the object had no or little effect on referent choices, a strong contextual cue toward the object resulted in a significant rise in object choices in both adults and children (aged 6;9–11;3). This was in stark contrast to their choices in complement control and *control* sentences, which remained uniform across every condition. Importantly, the fragility that adjunct control displayed in terms of its interpretation was also markedly different from pragmatically regulated control, as shown above in (8), which was also tested. In this instance, the cue determined referent choice definitively.

On the basis of these results, Janke and Bailey (2017) presented an analysis for this type of sentence-final adjunct control which could reflect these seemingly conflicting properties: Unlike complement control, which remains resilient to pragmatic cues, pragmatic cues preceding temporal adjunct control result in a significant number of children and adults adopting object interpretations. This generally only occurs under severe strong discourse pressure and not all participants are persuaded by the cue^2^. However, unlike controlled VGSs, interpretations in this type of adjunct control are restricted to within the sentence, cause nothing like the degree of interpretative shift seen in VGSs, and are renowned for not permitting generic interpretations—one of the hallmarks of a pragmatically regulated control relation. Thus, a structural analysis was proposed, which could account for the evident interpretation shift, yet not lose sight of the syntactic properties that this sub-type of control displays, namely the requirement that the *ec* has a sentence-internal, structurally dominant antecedent. Before we turn to the relevant sentences, note first that sentences with adjuncts are conventionally analyzed as having multiple attachment sites for the adjunct. This flexibility accounts for them not being restricted to a single interpretation, as illustrated in (11), where either the subject or the object can be linked to the prepositional phrase.

(11) The angry man chased the boy with a big stick.

When linked to the object, the adjunct attaches inside the VP within the domain of the object (see Larson, 2004) but when linked to the subject, the adjunct attaches higher, at the VP level, which is within the subject's structural domain. If we return now to sentence-final temporal adjunct control, a similar rationale can be used to account for the interpretations this construction permits. It is well established in the literature that the most popular interpretation of temporal adjunct control is one in which the subject is equated with the *ec*. On this parse, the adjunct adjoins at VP-level, as in (12), and only the subject c-commands into it so only a subject-oriented reading of the *ec* is possible.

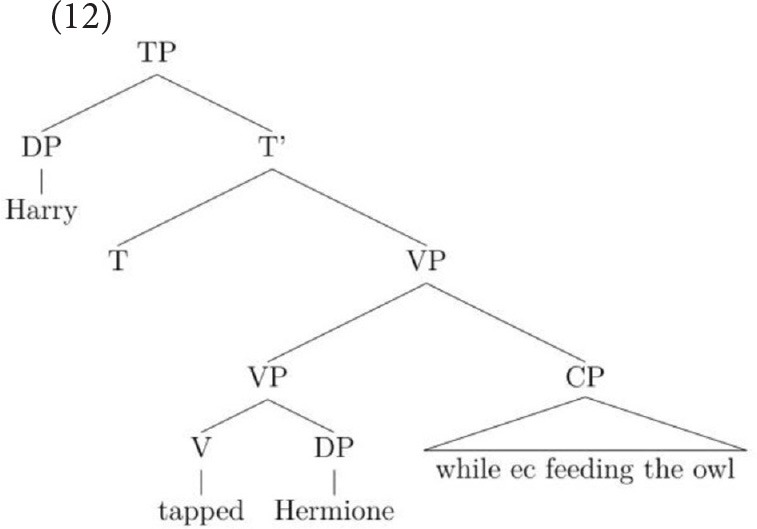

This structure accounts for the many people that prefer the subject-oriented reading but it cannot capture the grammar of speakers who permit an object-oriented reading under the discourse pressure generated by the strongly established topic. This is because the object does not c-command into the CP. However, by utilizing an analysis proposed independently for English VP structure in Janke and Neeleman (2012), Janke and Bailey (2017) proposed that speakers who allow an object-oriented reading permit the adjunct to attach low, merging directly with the verb, as in (13) (see also Larson, 2004). A consequence of this low attachment is that a VP-shell must be generated because in English, a verb must be left-adjacent to an argument that is dependent on it for accusative case (see Janke and Neeleman, 2012, for a full account). With a VP-shell configuration, both arguments c-command into the adjunct but the object is most local. On this parse, then, only an object reading of the *ec* is available.

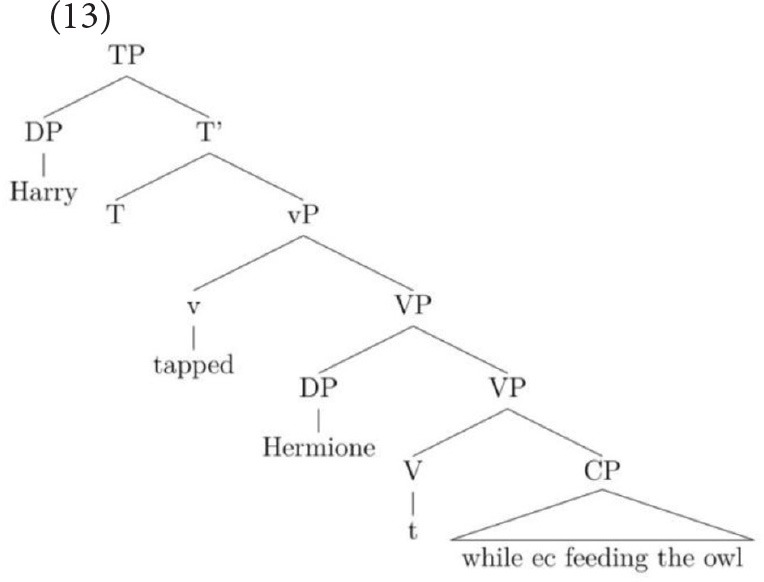

Note that it is because adjuncts allow more than one structure that this choice between two sentence-internal referents is possible: when the syntax provides more than one structural configuration, pragmatics can influence the way in which the string is parsed. In contrast, in complement control, only the VP-shell structure is available since control verbs select a CP that is obligatorily merged as the verb's complement (see Larson, 1991).

One important dimension to the temporal adjunct control data pattern is that these two interpretations are not equally favored. It therefore remains to account for why the subject parse is so much preferred over the object one. Janke and Neeleman (2012) argued that VP-shell formation is subject to a principle of economy, where a structure with no movement is more economical than one with movement:

(14) Economy
  - Two structures are in competition if and only if (i) they are well formed, and (ii) they are characterized by identical hierarchical relations, except for those hierarchical relations created by movement.
  - From a set of competing structures, choose the one with the fewest movements. (Janke and Neeleman, 2012: exx. (6))

The relevance of this analysis for current purposes is that a tree with no movement (i.e., no VP-shell) is more economical than a tree with movement (i.e., with a VP-shell) so the former structure should be highly preferred over the latter. Applying this to temporal adjunct control provides a means of modeling the data pattern observed, namely the overwhelmingly strong preference for subject interpretations. For full motivation of this account, the reader is referred to the original text. The important point for the current purposes is that it predicts subject-oriented adjunct control to be the highly preferred structure yet allows interpretations to change to the object under severe discourse pressure. In contrast, since complement control has an unambiguous structure, interpretations should not budge at all, and this is the precise pattern found in TD children and adults. It remains now for us to explore how HFA children perform on this construction, namely whether or not they will show the same initial preference for a subject interpretation, and whether preceding sentences that establish the matrix object as the topic of discourse will lead them to adopt the alternative, less economical, parse.

With the interpretative patterns of these three sub-types of control in place, we now return to what the current study will test and the outcomes that might be predicted from our children with ASD. With respect to complement control, we can formulate the following hypothesis:

Hypothesis 1
  If the syntax underlying complement control is unimpaired in HFA, all three groups' interpretations of the *ec* should pattern together, remaining uniformly object-oriented across the three conditions: the condition in which there is no cue, the condition in which the subject is cued by a weakly established topic and the condition in which the subject is cued by a strongly established topic.

Such a result would serve to further corroborate the previous studies' findings by replicating them. But of further importance is that it can contextualize our assessment of the children's attention to pragmatic cue in examples of control that are pragmatically regulated in adults, namely the controlled VGSs. If the children were persuaded by the topics in infelicitous circumstances (i.e., in complement control), then their liberal use of them in pragmatically regulated constructions would be less informative. If, however, they are ignoring topics when they are irrelevant, we have a clearer window through which to examine their pragmatic development.

Our predictions with regard to the controlled VGSs in the relevant conditions are as follows:

Hypothesis 2
  - If HFA children are performing typically with this construction, we expect a strong preference for the internal-referent in the no cue condition for all groups.
  - If HFA children consult the weak pragmatic cue in a typical way (where the weakly established topics cue either the internal or external referent), we expect all groups' referent choices to show a greater consensus for the referent that is being cued.
  - If HFA children consult the strong pragmatic cue in a typical way (where the strongly established topics cue either the internal or external referent), consensus for the cued referents should increase further for all groups.

Our predictions with regard to temporal adjunct control are more tentative. Using work on complex syntax in ASD and sentence-final temporal adjunct control in TD as a gauge, we can form the following predictions:

Hypothesis 3
  - If HFA children are performing typically with this construction, they should pattern with TD children and favor subject interpretations of the *ec* in the no cue condition.
  - If HFA children's attendance to the weak pragmatic cue is typical (where the weakly established topic cues the object), we should still find a higher level of consensus for a subject interpretation of the *ec* in all groups.
  - If HFA children's attendance to the strong pragmatic cue is typical (where the strongly established topic cues the object), we should now find an increased consensus for an object interpretation of the *ec* in all groups.

## Method

### Participants

This study was carried out in accordance with the recommendations of the “University of Kent's Research Ethics Committee,” with written informed consent from all participants. All participants gave written informed consent in accordance with the Declaration of Helsinki and the University of Kent's Research Ethics Committee approved this study (ID: 20101584).

Sixteen children (4 girls) aged 6-16, with a confirmed clinical diagnosis of ASD, attending primary and secondary schools in Kent and greater London were recruited for the study. Four children with ASD were excluded for not being able to complete the testing battery, while for one participant an incomplete experimental battery is available. No participants had any hearing impairments, neurological or genetic deficits and they were monolingual native English speakers. Two groups of children from Kent acted as control participants to the group with ASD, reported as typically developing by their respective schools' head teachers. One group was matched to the ASD group on the raw score of Matrices subtest of Kaufman Brief Intelligence Test (KBIT), TD KBIT group, and the other on the raw score of British Picture Vocabulary Scales 2 (BPVS-2), TD BPVS group. Details of each group's scores on standardized measures are given in Table 1.

**Table 1:** **Ages and mean scores (standard deviation) on tests of language and cognition for all age groups**.

	**ASD**	**SD**	**TD KBIT**	**SD**	**TD BPVS**	**SD**
CA in months	134.23	47.020	110.62	22.017	109.23	19.473
KBIT Raw	**28.62**	6.063	**28.15**	5.669		
KBIT SS	104.62	15.025	110.85	11.711		
BPVS Raw	**97.69**	21.975			**97.54**	21.137
BPVS SS	99.40	20.332			107.85	9.091
TROG Raw	14.85	3.625				
TROG SS	95.77	16.468				

*Measures in bold are those on which the groups were matched. SS, Standard Scores; KBIT, Kaufman Brief Intelligence Test, Matrices subtest; BPVS, British Picture Vocabulary Scales 2*.

### Materials

A two-choice picture-selection task in Janke (2016) and Janke and Perovic (2016) was employed. Four examples of control were included in the test battery but this report focuses on three: complement control, temporal adjunct control and controlled verbal gerund subjects^3^. For each trial, children were presented with two pictures and needed to choose the one that best matched the accompanying sentence. This appeared at the bottom of the screen whilst also presented auditorily through headphones. They were recorded in a sound-proof booth, using the voice of a native-speaking female researcher not involved with the project, who maintained a nuclear stress throughout. Item presentation was randomized automatically for each participant, and location of the correct picture was balanced throughout (left or right) as were the figures in the pictures. Four characters from the Harry Potter books (*Harry, Ron, Hermione*, and *Luna*) were used. In addition to the three critical sentence types, six *control* sentence sets were included. The first was a simple SVO sentence set, which checked that children could follow the reasoning of the task and the second was an SVO embedded sentence. The third tested knowledge of “while.” The fourth cued an incorrect interpretation of an SVO sentence with a weakly established topic, which tested whether children ignored a contextual cue for a sentence whose set interpretation is uncontroversial. The fifth cued an incorrect interpretation of an SVO sentence with a strongly established topic, which tested the same phenomenon but under still stronger pressure. Finally, the sixth tested understanding of cause relevant to the VGS condition. There were six trials in each condition, with three critical sentence types (complement control CC, adjunct control AC, and controlled verbal gerund subjects VGS) in three different conditions (no cue, weak cue, strong cue^4^) together with six *control* conditions (SVO, SVO_emb, while, SVO_WC, SVO_SC, cause), culminating in 102 sentences for each child. These sentences were distributed across three tasks, where they were divided according to the presence or absence of a cue: Task 1 presented the constructions with no cue, Task 2 preceded the constructions from Task 1 with a weakly established topic (weak cue), and Task 3 preceded them with a strongly established topic (strong cue). The simple of the task presentation was pseudo-randomized (more details in Section Procedure below).

### Sentence types

In this section, we illustrate examples of each construction tested, namely complement control, temporal adjunct control, controlled verbal gerund subjects, and the six *control* conditions. The complete set can be found in the Appendix (Supplementary Material).

For complement control, the matrix verbs were *persuade, simple* and *tell* and the verbs in the controlled clauses were *kick, mix* and *wave* respectively. The picture corresponding to the correct interpretation depicted the character represented by the matrix object engaged in an action, while the character represented by the matrix subject stood by. The foil showed the matrix subject engaging in the action. For the examples below, the corresponding picture showed *Ron* kicking the ball, with *Hermione* standing next to him, and the foil showed *Hermione* kicking the ball, with *Ron* standing next to her.

(14) Complement Control Test Sentence Examples
  - Hermione persuaded Ron *ec* to kick the ball.
  - Let me tell you something about Hermione. Hermione persuaded Ron *ec* to kick the ball.
  - Hermione is learning a new game. Hermione practises the rules. Hermione persuaded Ron *ec* to kick the ball.

For temporal adjunct control, the matrix verbs were *tap, kiss* and *lift* and the verbs in the controlled clause were *feed, fly* and *drink*. The picture corresponding to a subject interpretation of the *ec* depicted the character represented by the matrix subject engaged in an action, while the character represented by the matrix object stood by. In the alternative picture, the matrix object engaged in the action. For the sentences below, the picture aligned with a subject interpretation had *Harry* tapping *Luna* with *Harry* feeding the owl, and the picture aligned with an object reading had *Harry* tapping *Luna* with *Luna* feeding the owl.

(15) Temporal Adjunct Control Test Sentence Examples
  - Harry tapped Luna while *ec* feeding the owl.
  - Let me tell you something about Luna. Harry tapped Luna while *ec* feeding the owl.
  - Luna is looking after the birds. Luna takes out the food. Harry tapped Luna while *ec* feeding the owl.

For controlled VGSs, the main verbs used were *pour, read* and *row*.

(16) a. *ec* Reading the book slowly made Hermione sleepy.
     b. Let me tell you something about Ron. *ec* Reading the book slowly made Hermione sleepy.
     c. Ron is looking up a spell. Ron says each word carefully. *ec* Reading the book slowly made Hermione sleepy.

For the first *control* condition, which was an SVO sentence in the progressive, the corresponding picture showed the subject engaged in the activity, whereas the foil depicted an unmentioned character as the agent. In the example below, the correct picture showed *Harry* mixing the flour with *Hermione* standing next to him and the foil showed the reverse.

(17) SVO *Control* Sentence Example
         Harry is mixing the flour.

In the “while” *control* condition, as illustrated in (18), the corresponding picture showed both characters engaging in the actions described. In the foils, only one of the characters is engaged in the relevant activity while the other stands by passively. For half the trials, the character not meeting the description was in the main clause and for the other half this was the character in the embedded clause.

(18) While *Control* Sentence Example
         Hermione is feeding the owl while Harry is waving the wand.

The *control* condition for the weakly established topic consisted of an embedded SVO sentence preceded by a weakly established topic. In the correct picture for (19), *Ron* is drinking the potion and *Hermione* is standing next to him. In the foil, *Hermione* is drinking the potion.

(19) Weakly Established Topic SVO *Control* Sentence Example
         Let me tell you something about Hermione. Hermione said that Ron is drinking the potion.

The *control* condition for the strongly established topic preceded an SVO sentence with a strongly established topic. For (20), in the correct picture, *Harry* is waving the wand with *Luna* standing nearby and in the foil, the reverse occurs.

(20) Strongly Established Topic SVO *Control* Sentence Example
         Luna is learning a difficult spell for a class test. Luna says the magic words slowly. Harry is waving the wand.

The fifth *control* sentence was applicable to VGS in that it tested understanding of causation such as in (21). In the correct picture, *Hermione* was pouring water and spilling it over herself with *Ron* standing by, whereas in the foil, *Ron* was pouring the water and spilling it on *Hermione*.

(21) The water made Hermione wet.

Finally, an embedded SVO *control* sentence was included. In the correct picture the subject of the embedded clause was engaged in the action (*Ron* in the example below) and in the foil, the matrix subject (*Hermione*) was the agent of the activity.

(22) Hermione said that Ron is drinking the potion.

### Procedure

Administration of the three tasks and the standardized assessments (BPVS II; KBIT; TROG-2) occurred over three testing sessions, each lasting between 30 and 40 min. BPVS II, KBIT and the first experimental task were administered in the first session, whereas in the second and third session, the simple of the second and third experimental task was randomized for each child. TROG was administered either in the second or third session. For participants with ASD, if the child showed poor performance on *control* conditions (e.g., SVO, SVO_embedded) in the first experimental task, the remaining experimental tasks were not administered and the child's data were not included in the analysis; this was the case for four children.

Experimental stimuli were presented on a laptop and randomized by computer software. Prior to the trial, children were shown pictures of the characters engaged in various activities and told their names. They were asked to point to each of the characters the experimenter named and to identify various activities occurring in the pictures, for example, “Show me Luna is popping the balloon” and “Show me Ron is reading the book.” All the children succeeded with this phase. They were then told that they would be shown two pictures and see and hear a sentence describing the pictures. After the sentence had finished playing, they needed to choose the picture they thought went best with the sentence. The children made their choice by clicking on one of the large tabs by each picture, which appeared once the sentence had played, preventing them from making a premature choice. The children received a book voucher as a ‘thank you’ for taking part.

## Results

### Results on the *control* conditions

All children performed at ceiling on the *control* conditions (see Table 2). These scores were not analyzed further due to ceiling effects.

**Table 2:** **Mean correct responses in the ***control*** conditions**.

	**ASD**	**TD KBIT**	**TD BPVS**
SVO	0.99	1.00	1.00
SVO embedded	0.93	0.99	0.94
SVO weak cue	1.00	1.00	1.00
SVO strong cue	0.96	0.99	1.00
“cause”	1.00	0.99	1.00
“while”	0.99	1.00	1.00

### Results on complement control (CC) and temporal adjunct control (AC)

A generalized linear mixed model (GLMM) executed in SPSS 22 was used to analyse the data for the CC and AC constructions. Fixed effects entered into the model were Group (ASD, TD_KBIT, TD_BPVS), Construction (CC and AC), Condition (no cue, weak cue and strong cue), and the Group^*^Condition^*^Construction interaction.

The model showed significant main effects of Construction *F*_(1, 1, 338)_ = 422.45, *p* < 0.001, Condition *F*_(2, 1, 338)_ = 6.066, *p* = 0.002, and a significant Group^*^Condition^*^Construction interaction: *F*_(12, 1, 338)_ = 3.841, *p* < 0.001. The main effect of Group was not significant: *F*_(2, 1, 338)_ = 0.275, *p* = 0.759.

**Figure 1:**
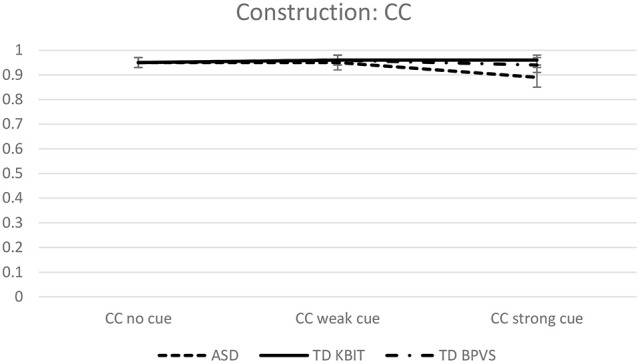
**Estimated mean object responses on CC across all conditions**.

**Figure 2:**
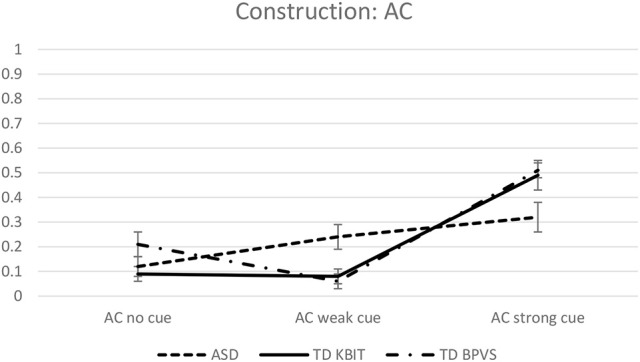
**Estimated mean object responses on AC across all conditions**.

Estimated mean object responses for each group on the two constructions, across three conditions, are given in Figures 1, 2, revealing strikingly different patterns on CC vs. AC for each of three groups.

On the CC construction, Sidak-corrected pairwise comparisons included in the model revealed no statistically significant group differences in any of the three conditions (no cue, weak cue or strong cue). In contrast, the groups showed different performance on the AC construction on some of the conditions (estimated mean responses for an easy comparison are given in Table 3). On the *no cue* condition, no difference between groups was observed: ASD vs. TD_KBIT *t*_(1, 338)_ = 0.522, *p* = 0.602; ASD vs. TD_BPVS *t*_(1, 338)_ = 1.5221, *p* = 0.240. On the *weak cue* condition, the difference between the ASD group and both control groups was significant: TD_KBIT *t*_(1, 338)_ = 2.526, *p* = 0.023 and TD_BPVS *t*_(1, 338)_ = 2.973, *p* = 0.009. On the *strong cue* condition, the differences almost reached statistical significance: ASD vs. TD_KBIT *t*_(1, 338)_ = 2.099, *p* = 0.071 and ASD vs. TD_BPVS *t*_(1, 338)_ = 2.381, *p* = 0.051. Comparisons of the performance of the two control groups, TD_KBIT vs. TD_BPVS, revealed no differences on any of the cues: *no cue*: *t*_(1, 338)_ = 2.035, *p* = 0.121; *weak cue*: *t*_(1, 338)_ = 0.449, *p* = 0.654; *strong cue*: *t*_(1, 338)_ = 0.321, *p* = 0.748.

**Table 3:** **Estimated mean object responses on AC and CC across all conditions**.

	**ASD**	**TD KBIT**	**TD BPVS**
AC no cue	0.12	0.09	0.21
AC weak cue	0.24	0.08	0.06
AC strong cue	0.32	0.49	0.51
CC no cue	0.95	0.95	0.95
CC weak cue	0.95	0.96	0.96
CC strong cue	0.89	0.96	0.94

To get a better picture of within group effects, within group Sidak-corrected comparisons were carried out for each of the two constructions and across the three conditions. On the CC construction, none of the groups revealed differences between their performance on the three different conditions—no cue, weak cue and strong cue. However, on the AC construction, there were significant within-group differences on individual conditions. All three groups showed a difference between *no cue* vs. *strong cue* on AC (*p* = 0.006 for ASD, and *p* < 0.001 for the two control groups). The ASD group showed no other differences: their performance on no cue vs. weak cue was not significantly different, neither was weak cue vs. strong cue. The TD_KBIT group showed a difference between weak vs. strong cue (*p* < 0.001), but not between no cue vs. weak cue. The TD_BPVS group showed a difference between all different cues: no cue vs. weak cue (*p* = 0.009) and weak and strong cue (*p* < 0.001).

### Results on verbal gerund subjects

The second GLMM analysis was run to examine the groups' performance on the VGS constructions, in 5 conditions, with Group (ASD, TD_KBIT and TD_BPVS) and Condition (No cue, Weak Cue Internal Referent, Weak Cue External Referent, Strong Cue Internal Referent, Strong Cue External Referent), and the Group^*^Condition interaction included in the model.

**Figure 3:**
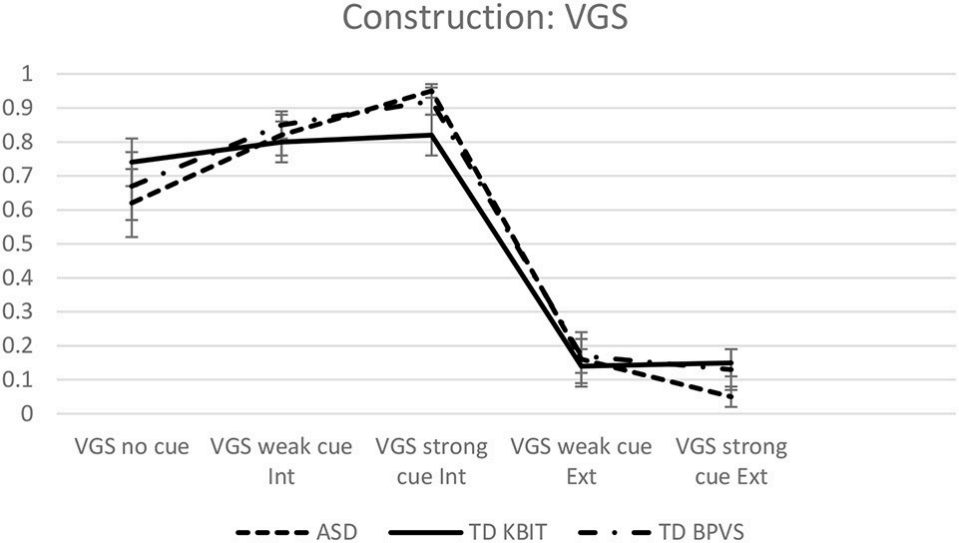
**Estimated mean internal referent responses on VGS across all conditions**.

The model showed no statistically significant effect of Group *F*_(2, 40)_ = 0.209, *p* = 0.813, a highly significant effect of Condition *F*_(4, 195)_ = 47.176, *p* < 0.001 and a significant Group^*^Condition interaction, *F*_(8, 242)_ = 2.732, *p* = 0.007. The estimated mean internal referent responses for each group across the five conditions are shown in Figure 3.

Pairwise Sidak-corrected analyses included in the model showed no significant differences between the performances of any of the three groups on any of the five conditions.

However, within-group comparisons of participants' performance on the five conditions revealed a larger number of significantly different comparisons in the ASD group, and a smaller number of significant comparisons in the two control groups, which is what drove the significant Group^*^Condition interaction. In the ASD group, except for the non-significant comparison of weak cue External Referent vs. strong cue External referent [*t*_(86)_ = 1.340, *p* = 0.184], children's performance on all other conditions was significantly different when compared to other conditions; (see Table 4), with *p*-values ranging from *p* = 0.024 to *p* < 0.001.

**Table 4:** **Estimated mean internal referent responses on VGS across all conditions**.

	**ASD**	**TD KBIT**	**TD BPVS**
VGS no cue	0.62	0.74	0.67
VGS weak cue Int	0.82	0.80	0.85
VGS strong cue Int	0.95	0.82	0.92
VGS weak cue Ext	0.16	0.14	0.17
VGS strong cue Ext	0.05	0.15	0.13

*Int, Internal Referent; Ext, External Referent*.

In the TD_BPVS group, again children' performance on no cue sentences was not significantly different when compared to weak or strong cue sentences involving Internal Referent, but was highly significantly different when compared to sentences involving External Referent, under both the weak cue [*t*_(532)_ = 9.784, *p* < 0.001] and strong cue [*t*_(197)_ = 7.344, *p* < 0.001].

The weak cue Internal Referent did not differ to strong cue Internal Referent [*t*_(849)_ = 0.505, *p* = 0.851], but performance on sentences involving Internal Referent was highly significantly different to all performances on sentences involving External Referent: weak cue Internal Referent vs. weak cue External Referent [*t*_(1, 131)_ = 12.397, *p* < 0.001], weak cue Internal Referent vs. strong cue External Referent [*t*_(250)_ = 9.176, *p* < 0.001]; strong cue Internal Referent vs. weak cue External Referent [*t*_(673)_ = 11.587, *p* < 0.001], and strong cue Internal Referent vs. strong cue External Referent [*t*_(185)_ = 10.198, *p* < 0.001]. The two External Referent conditions did not differ when compared to each other: weak cue External Referent vs. strong cue External Referent: [*t*_(308)_ = 0.193, *p* = 0.851].

In the TD_BPVS group, again the no cue sentences did not differ to sentences involving Internal Referent, but was highly significantly different when compared to sentences involving External Referent, under both the weak cue [*t*_(73)_ = 4.596, *p* < 0.001] and strong cue [*t*_(68)_ = 4.420, *p* < 0.001].

Performance on sentences involving Internal Referent did not differ from each other: weak cue Internal Referent vs. strong cue Internal Referent [*t*_(228)_ = 1.245, *p* = 0.383], but performance on sentences involving Internal Referent was highly significantly different to all performances on sentences involving External Referent: weak cue Internal Referent vs. weak cue External Referent [*t*_(596)_ = 16.036, *p* < 0.001], weak cue Internal Referent vs. strong cue External Referent [*t*_(291)_ = 12.926, *p* < 0.001]; strong cue Internal Referent vs. weak cue External Referent [*t*_(139)_ = 11.036, *p* < 0.001] and strong cue Internal Referent vs. strong cue External Referent [*t*_(124)_ = 10.908, *p* < 0.001]. The group's performance on the two External Referent conditions did not differ: weak cue External Referent vs. strong cue External Referent: [*t*_(1, 000)_ = 1.041, *p* = 0.383).

## Discussion

The aim of this study was to establish whether high-functioning children with autism respond differently to non-verbal and verbal MA-matched TD children when presented with contextual cues of different strengths on three sub-types of control: complement control, controlled verbal- gerund subjects and sentence-final temporal adjunct control. There were several main findings. First, children in all three groups demonstrated the same resilience to weakly and strongly established discourse topics in complement control. That is, they opted for the object interpretation consistently across all three conditions. Second, the HFA children's attendance to the topics in controlled VGSs was very similar to that of the TD groups. All three groups' referent choices were influenced by the topics. In the no cue condition, all groups showed a preference for the sentence-internal referent, however, this preference was stronger in the TD groups, which meant the HFA group had significantly fewer internal-referent responses than the typical groups in this condition. The weakly established topics generally had a very strong effect on all children's interpretations, whose referent choices were largely determined by the cue. The decisive influence of this weak cue meant that the effect of the strongly established topic was masked. The result was that in most cases, there was no further shift toward the cued referent in this condition. Third, the results for sentence-final temporal adjunct control showed the groups to be behaving very similarly in one respect yet slightly differently in another. In the no cue condition, the three groups performed on a par with each other, all illustrating overwhelming consensus for a subject-oriented interpretation of the *ec*. In the condition that used a weakly established topic to cue the object, the TD groups' object choices remained stable relative to the choices made in the no cue condition, whilst the HFA group showed a small increase in accepting the object choices. In the condition that used a strongly established topic to cue the object, all groups' object choices increased significantly, yet the increase in the HFA group was smaller, resulting in the TD groups' number of object choices being somewhat greater than the HFA group's number of object choices, as illustrated in Figure 1.

We begin our discussion with the *control* items, before progressing to complement control and controlled VGSs, where we will indicate how these results relate to earlier literature on HFA children's performances on these constructions. After this, we turn to temporal adjunct control, where a number of possible explanations for these results are discussed.

Firstly, all children's performances on the *control* conditions were at ceiling. This meant that they could understand the task, they could comprehend embedded sentences, they understood the meaning of “while” entailed that two people engaged in an action simultaneously, and the basic cause-effect relation described in the VGS sentences—all over the course of three testing sessions lasting at least 25 min each. In addition, the conditions including pragmatic leads demonstrated that children could ignore infelicitous cues for sentences whose references are set.

Turning to complement control, we saw that there were no differences between the clinical and typical groups. All three groups, therefore, recognized the obligatory syntactic relation between the *ec* in the controlled complement and the object in the matrix clause. These results support the two earlier aforementioned studies on two different groups of HFA children (Janke and Perovic, 2015, 2016), both of whom gave object choices uniformly, too. Three studies culminating in the same pattern of results strongly support our argument that this example of syntax is unimpaired in HFA. The contribution of these results, namely that complement control has proven resilient to infelicitous cues, enables us to probe children's proficiency of pragmatically regulated constructions, confident that children are able to discern between terms whose references are regulated syntactically and terms whose references require attention to the context for their resolution.

Our next question was whether the HFA children's attention to contextual cues differs to that of TD children when assigning reference to the *ec*s in controlled VGSs. Firstly, in the no cue condition, although all groups demonstrated a preference for the internal referent, this preference was less pronounced in the HFA group, particularly in comparison to the TD-KBIT group. This result is important as it might answer for the subtle differences between the populations in the subsequent conditions. Turning to the cueing of the internal referent first, when this was cued by a weakly established topic, the HFA group's internal referent choices rose significantly. When cued by a strongly established topic in this same direction, the HFA group's internal referent choices increased significantly once again. In the other two groups, however, although interpretations could be seen to shift (recall Table 4), the topics did not significantly raise internal-referent choices from the baseline. This difference in the cues' effects could be seen as a product of the HFA children's initial lower number of internal-referent choices, which allowed the cues to come into effect. With respect to the conditions which cued the external referent, all three groups showed the same pattern. In the condition which cued the external referent with a weakly established topic, all groups' internal referent choices decreased dramatically—so much so that the effect of this cue was strong enough to mask any influence of the strongly established topic. In this latter condition, internal referent choices did not decrease further for any of the groups. On this basis, we can conclude that the populations are responding in a remarkably similar way to the pragmatic leads. The results are also in line with those reported in Janke and Perovic (2016), where that HFA population also showed no difference in performance on this construction from their matched TD controls.

At this point, we have distinguished between HFA children's responses in two types of control, one of which is syntactically regulated, the other of which is pragmatically regulated. In both cases, children performed on a par with the TD children. The one difference between the TD and HFA children can be sourced to the HFA children's slightly lower level of consensus for an internal referent in the no cue condition than the TD children so the hypothesis that HFA children would attend to the cue in a way that is not markedly different to TD on either of these constructions can be upheld.

The final construction we turn to is sentence-final temporal adjunct control. Recall that this construction is not a proto-typical example of obligatory control but neither does it have the signature properties of a pragmatically regulated type. Unlike in complement control, where the complement is sister to the verb, the adjunct is not selected by the verb. However, the *ec* in sentence-final temporal adjunct control does not permit external referents, unlike pragmatically regulated control relations. Let us first consider why all three groups of children's interpretations might have shifted from the baseline at all. Sentence-final temporal adjunct control has long been analyzed as strictly subject-oriented (see Landau, 2013) so the current results might not have been predicted to have occurred. However, the introduction discussed recent experimental work on this sub-type of adjunct control which revealed that children's and adults' interpretations of the *ec* are not in fact uniformly subject-oriented. To recap, it showed that the same paradigms had demonstrated that a strong pragmatic cue toward the object resulted in a significant consensus for object choices in adults and children aged from 6 to 11. Importantly, this pattern of results was very different from complement control (which remained unaffected by the cue) and also from VGSs (which were affected uniformly by the cues), thereby motivating an alternative account for this type of adjunct control. Specifically, it was shown how an independently motivated analysis of English VP structure (Larson, 2004; Janke and Neeleman, 2012) could be employed to account for the interesting data pattern that had emerged from the TD children and adults: The most economical structure was one where no VP-shell had been generated. This should, therefore, be the highly preferred structure when all else is equal. When the tree is parsed in this way, only the subject interpretation is possible, as in (23), and this is indeed the highly preferred interpretation.

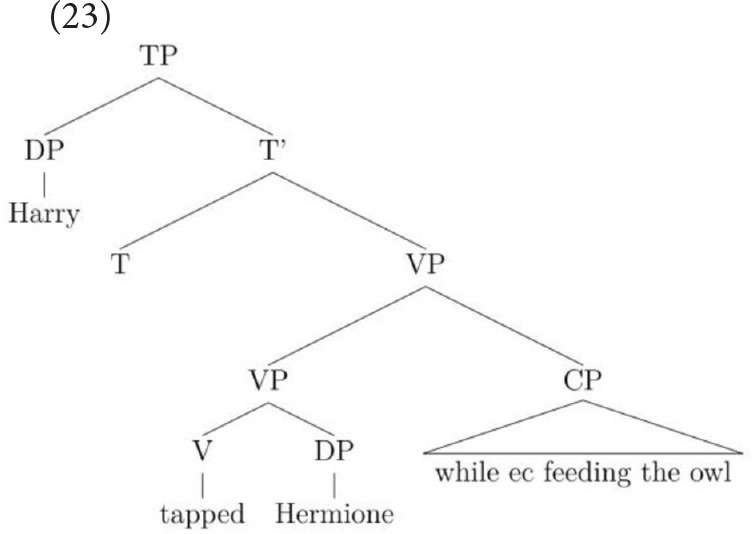

Under severe discourse pressure, however, such as that generated by a strongly established topic, an alternative parse is licit on this account. This less economical parse gives rise to a VP-shell, which leaves the object as the most local c-commanding antecedent of the *ec*, as repeated in (24). On this parse, only an object interpretation is syntactically licit, representing the TD and adult participants' switch to the object in this strongly cued condition.

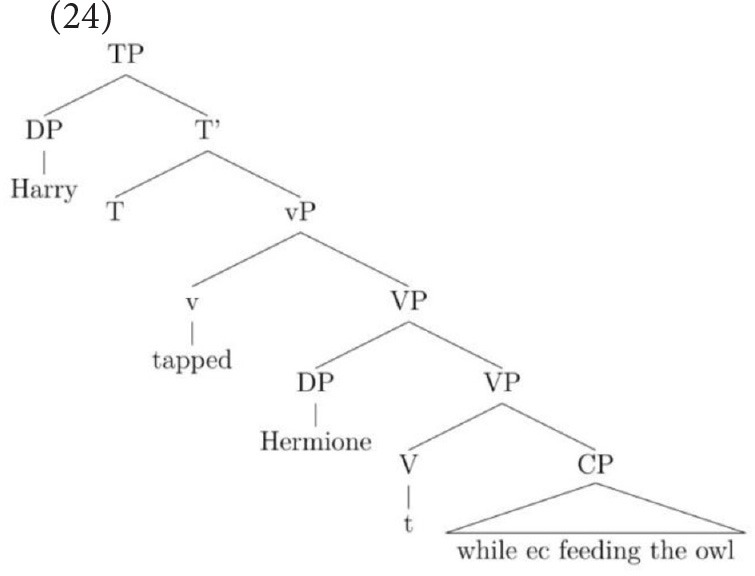

If we return now to the current children's preferences in the adjunct control sentences which contained no cue, we can note that all three groups displayed the above pattern: they all showed a strong preference for the subject in the no cue condition, thereby adopting the most economical, and so highly preferred, parse. In the second condition that employed the weakly established topic, children with ASD already started to pay some attention to the cue, whereas TD groups still ignored it. In the third condition, all groups showed a significant shift toward the object—indicating that all three groups were consulting the cue - though the degree to which the cue was effective was slightly different: the HFA group's object choices were somewhat lower than those of the two TD control groups'. The pattern of a gradual rather than a sudden increase in the HFA group across different strength of the cue is a result which now needs to be replicated in a further study, but, crucially, indicates that children with ASD do consider these contextual cues in their interpretation of sentence-final adjunct control.

To conclude, given the widely reported pragmatic and syntactic deficits in populations with ASD, the relatively straightforward patterns observed in our sample of children point to similarities, rather than differences, in the linguistic profiles of high-functioning children with ASD and their matched TD controls. In regard to this last construction in particular, it is important to note that there are a number of typical adults and children who are reticent to abandon their initial subject interpretations under the same level of discourse pressure. The subtle difference found in children's interpretations of this construction, therefore, does not in itself warrant an appeal to the hypothesized reduced cognitive flexibility reported in the literature, in line with Geurts et al. (2009). Our study, the first to compare the three sub-types of control in ASD in the literature, reveals that, in relevant contexts, HFA children consult the pragmatic cues similarly to TD children, despite diverse pragmatic deficits reported for this population, suggesting that (at least certain aspects of) primary pragmatics are functioning well in this ASD sub-group.

## Author contributions

VJ and AP conceived the study. VJ and AP shared the data collection. VJ collated and transcribed the data. AP analyzed the data and wrote the results section. VJ wrote the introduction, the method and the discussion. Both authors edited the final version of the manuscript and have agreed to be accountable for the content of the manuscript.

## Funding

The study was piloted and experimental materials were developed with funds from a British Academy Small Research Grant (SG112896).

### Conflict of interest statement

The authors declare that the research was conducted in the absence of any commercial or financial relationships that could be construed as a potential conflict of interest.

## Footnotes

^1^There is a well-known exception in double-complement control, namely the double-complement *subject* control construction, exemplified most often by the verb “promise,” where the subject controls the *ec* (John_1_ promised Peter *ec*_1_ to write the letter). This construction is acquired late (Chomsky, 1969) and is not fully accepted by all speakers (see Janke and Perovic, 2015, for a first study of HFA children's interpretation of this construction). In this article, we focus only on double-complement *object* control.

^2^Interestingly, even in the “no cue” condition, complement control and temporal adjunct control result in slightly different outcomes: whereas complement control proves to be resolutely object-oriented, responses in temporal adjunct control, although predominantly subject-oriented, do display some variation. There is also a minority of adults that demonstrates a preference for an object-oriented reading in this base-line condition.

^3^Another type of non-obligatory control tested in the same battery is reported on in separate work.

^4^Note that the VGS construction was cued in two directions in both cued conditions, namely toward the external referent and towards the external referent. This means there are 12 trials for this construction in each of these conditions, not 6.

## Abbreviations

AC: sentence-final temporal adjunct control
ALN: autism language normal
ASD: autism spectrum disorders
BPVS: British Picture Vocabulary Scales
CA: chronological age
CC: complement control
HFA: high functioning autism
KBIT: Kaufman Brief Intelligence Test
MA: mental age
RS: raw score
SS: standard score
TD: typical development or typically developing
TROG: Test of Reception of Grammar
VGS: controlled verbal-gerund subject.
