# Supplementary material for: Contrasting Complement Control, Temporal Adjunct Control and Controlled Verbal Gerund Subjects in ASD: The Role of Contextual Cues in Reference Assignment
Source: Front Psychol. 2017 Mar 28;8:448. doi: 10.3389/fpsyg.2017.00448 (PMC5369323; doi:10.3389/fpsyg.2017.00448)
Supplement: Supplementary file 1 [file DataSheet1.docx]

**Appendix. Test Sentences and *Control* Sentences**

**(NO CUE)**

**Complement Control Test Sentences**

Hermione ordered Harry to mix the flour.

Harry ordered Hermione to mix the flour.

Ron persuaded Hermione to kick the ball.

Hermione persuaded Ron to kick the ball.

Luna told Harry to pop the balloon.

Harry told Luna to pop the balloon.

**Temporal Adjunct Control Test Sentences**

Ron kissed Hermione while flying the broom.

Hermione kissed Ron while flying the broom.

Harry tapped Hermione while feeding the owl.

Hermione tapped Harry while feeding the owl.

Ron lifted Luna while drinking the potion.

Luna lifted Ron while drinking the potion.

**Verbal Gerund Subject Sentences**

Pouring the water quickly made Luna wet.

Pouring the water quickly made Harry wet.

Rowing the boat clumsily made Ron seasick.

Rowing the boat clumsily made Luna seasick.

Reading the book slowly made Hermione sleepy.

Reading the book slowly made Ron sleepy.

**SVO *Control* Sentences**

Hermione is feeding the owl.

Harry is lifting the book.

Hermione is kicking the ball.

Ron is rowing the boat.

Harry is mixing the flour.

Luna is kissing the owl.

**While *Control* Sentences**

Hermione is feeding the owl while Harry is waving the wand.

Harry is feeding the owl while Hermione is waving the wand.

Luna is flying the broom while Harry is lifting the book.

Harry is flying the broom while Luna is lifting the book.

Luna is kissing the owl while Ron is popping the balloon.

Ron is kissing the owl while Luna is popping the balloon.

**Cause *Control* Sentences**

The book made Ron sleepy.

The book made Hermione sleepy.

The potion made Harry wet.

The potion made Hermione wet.

The boat made Ron seasick.

The boat made Luna seasick.

**(WEAK CUE: Weakly Established Topic)**

**Complement Control Test Sentences**

Let me tell you something about Hermione. Hermione ordered Harry to mix the flour.

Let me tell you something about Harry. Harry ordered Hermione to mix the flour.

Let me tell you something about Ron. Ron persuaded Hermione to kick the ball.

Let me tell you something about Hermione. Hermione persuaded Ron to kick the ball.

Let me tell you something about Luna. Luna told Harry to pop the balloon.

Let me tell you something about Harry. Harry told Luna to pop the balloon.

**Temporal Adjunct Control Test Sentences**

Let me tell you something about Hermione. Ron kissed Hermione while flying the broom.

Let me tell you something about Ron. Hermione kissed Ron while flying the broom.

Let me tell you something about Hermione. Harry tapped Hermione while feeding the owl.

Let me tell you something about Harry. Hermione tapped Harry while feeding the owl.

Let me tell you something about Luna. Ron lifted Luna while drinking the potion.

Let me tell you something about Ron. Luna lifted Ron while drinking the potion.

**Verbal Gerund Subject Test Sentences (External/Internal Referent Cued)**

Let me tell you something about Luna/Harry. Pouring the water quickly made Harry wet.

Let me tell you something about Luna/Harry. Pouring the water quickly made Luna wet.

Let me tell you something about Ron/Hermione. Reading the book slowly made Ron sleepy.

Let me tell you something about Ron/Hermione. Reading the book slowly made Hermione sleepy.

Let me tell you something about Ron/Luna. Rowing the boat clumsily made Luna seasick.

Let me tell you something about Ron/Luna. Rowing the boat clumsily made Ron seasick.

**SVO Embedded *Control* Sentences**

Let me tell you something about Ron. Ron said that Hermione is feeding the owl.

Let me tell you something about Luna. Luna said that Harry is waving the wand.

Let me tell you something about Harry. Harry said that Luna is pouring the water.

Let me tell you something about Hermione. Hermione said that Harry is mixing the flour.

Let me tell you something about Ron. Ron said that Luna is rowing the boat.

Let me tell you something about Hermione. Hermione said that Ron is drinking the potion.

**(STRONG CUE: Strongly Established Topic)**

**Complement Control Test Sentences**

Hermione is having a party. Hermione prepares all the food. Hermione ordered Harry to mix

the flour.

Harry is having a party. Harry prepares all the food. Harry ordered Hermione to mix the

flour.

Ron is learning a new game. Ron practises the rules. Ron persuaded Hermione to kick the

ball.

Hermione is learning a new game. Hermione practises the rules. Hermione persuaded Ron to

kick the ball.

Luna is performing a new trick. Luna takes out the pin. Luna told Harry to pop the balloon.

Harry is performing a new trick. Harry takes out the pin. Harry told Luna to pop the balloon.

**Temporal Adjunct Control Test Sentences**

Hermione is preparing for a competition. Hermione practises in the air. Ron kissed Hermione

while flying the broom.

Ron is preparing for a competition. Ron practises in the air. Hermione kissed Ron while

flying the broom.

Hermione is looking after the birds. Hermione takes out the food. Harry tapped Hermione

while feeding the owl.

Harry is looking after the birds. Harry takes out the food. Hermione tapped Harry while

feeding the owl.

Luna is preparing an invisibility spell. Luna holds up the goblet. Ron lifted Luna while

drinking the potion.

Ron is preparing an invisibility spell. Ron holds up the goblet. Luna lifted Ron while drinking

the potion.

**Verbal Gerund Subject Test Sentences (Internal/External Referent Cued)**

Ron/Hermione is looking up a spell. Ron/Hermione says each word carefully. Reading the book

slowly made Ron sleepy.

Ron/Hermione is looking up a spell. Ron/Hermione says each word carefully. Reading the book

slowly made Hermione sleepy.

Hermione/Harry is making a potion. Hermione/Harry holds the cup clumsily. Pouring the water

quickly made Hermione wet.

Hermione/Harry is making a potion. Hermione/Harry holds the cup clumsily. Pouring the water

quickly made Harry wet.

Luna/Ron is going out on the lake. Luna/Ron takes the oars awkwardly. Rowing the boat clumsily

made Ron seasick.

Luna/Ron is going out on the lake. Luna/Ron takes the oars awkwardly. Rowing the boat clumsily

made Luna seasick.

**SVO *Control* Sentences**

Ron is looking after the birds for the day. Ron puts the food into the bowl. Hermione is

feeding the owl.

Luna is learning a difficult spell for a class test. Luna says the magic words slowly. Harry is

waving the wand.

Harry is making a magic potion in front of the whole class. Harry lifts up the yellow cup. Luna is

pouring the water.

Hermione is inviting the whole class to a birthday party. Hermione prepares a beautiful

chocolate cake. Harry is mixing the flour.

Ron is taking a trip out onto Hogwarts lake. Ron takes hold of the wooden oars. Luna is

rowing the boat.

Hermione is mixing the ingredients for a spell. Hermione takes up the small blue goblet.

Ron is drinking the potion.
